# Screening obstructive sleep apnea patients via deep learning of knowledge distillation in the lateral cephalogram

**DOI:** 10.1038/s41598-023-42880-x

**Published:** 2023-10-18

**Authors:** Min-Jung Kim, Jiheon Jeong, Jung-Wook Lee, In-Hwan Kim, Jae-Woo Park, Jae-Yon Roh, Namkug Kim, Su-Jung Kim

**Affiliations:** 1 Department of Convergence Medicine, Asan Medical Institute of Convergence Science and Technology, Asan Medical Center, University of Ulsan College of Medicine, 88 Olympic-ro 43-Gil Songpa-gu, Seoul, 05505 Republic of Korea; 2 Department of Biomedical Engineering, Asan Medical Institute of Convergence Science and Technology, Asan Medical Center, College of Medicine, University of Ulsan, Seoul, Republic of Korea; 3 Department of Orthodontics, Kyung Hee University Dental Hospital, Seoul, 05505 Republic of Korea; 4 Department of Radiology, University of Ulsan College of Medicine, Asan Medical Center, 88 Olympic-Ro 43-Gil Songpa-Gu, Seoul, 05505 Republic of Korea; 5 Department of Orthodontics, School of Dentistry, Kyung Hee University, 23, Kyungheedae-ro, Dongdaemun-gu, Seoul, 02447 Republic of Korea

**Keywords:** Computer science, Scientific data

## Abstract

The lateral cephalogram in orthodontics is a valuable screening tool on undetected obstructive sleep apnea (OSA), which can lead to consequences of severe systematic disease. We hypothesized that a deep learning-based classifier might be able to differentiate OSA as anatomical features in lateral cephalogram. Moreover, since the imaging devices used by each hospital could be different, there is a need to overcome modality difference of radiography. Therefore, we proposed a deep learning model with knowledge distillation to classify patients into OSA and non-OSA groups using the lateral cephalogram and to overcome modality differences simultaneously. Lateral cephalograms of 500 OSA patients and 498 non-OSA patients from two different devices were included. ResNet-50 and ResNet-50 with a feature-based knowledge distillation models were trained and their performances of classification were compared. Through the knowledge distillation, area under receiver operating characteristic curve analysis and gradient-weighted class activation mapping of knowledge distillation model exhibits high performance without being deceived by features caused by modality differences. By checking the probability values predicting OSA, an improvement in overcoming the modality differences was observed, which could be applied in the actual clinical situation.

## Introduction

Obstructive sleep apnea (OSA) is a complex and heterogeneous disorder that draws attention worldwide, because undetected OSA can lead to consequences of severe systematic disease^1,2^, heart disease^3^, cardiovascular dysfunction^4^, stroke^5^, and even sudden death^6^. Therefore, diagnosing OSA as early as possible takes precedence for the proper management of OSA^7^. Polysomnography (PSG) is regarded as the gold standard method used for OSA diagnosis^8^. Nevertheless, PSG is an overnight examination that is expensive and requires high patient compliance; additionally, it has a high risk of invalid study results^9,10^.

One of the major phenotypic causes of OSA is craniofacial structural abnormality^11,12^. The predisposing characteristics of OSA on the craniofacial structures are a narrowed posterior airway space, a long, elongated pharynx, thicker soft palate, a long and large tongue, a lower position of the hyoid bone, increased anterior lower facial height, and decreased sagittal dimension of the cranial base. The lateral cephalogram has been acknowledged as the tool confirming the potential relevance of OSA in patients with suspected symptoms^13–15^.

Deep learning is a subset of artificial intelligence (AI) technique that can learn from special features and make predictions about image data with or without supervision^11^. Amongst the various deep learning approaches, convolutional neural networks (CNNs) have been highlighted in image recognition to recognize anatomical structures in medical images automatically^12^. Savoldi et al.^13^ applied lateral cephalograms to evaluate the anatomical structures associated with OSA in children. They reported a limited reliability in the assessment of the tongue and soft palate area using lateral cephalograms. Tsuiki et al.^14^ included groups of patients with severe OSA and non-OSA according to the craniofacial morphology. They employed the deep learning approach to perform lateral cephalogram-based image classification, with successful identification of individuals with severe OSA by deep CNNs using lateral cephalogram.

Various modalities in cephalograms have become vital in the actual clinical setting^15^. Therefore, robust training in multimodal imaging has drawn attention in the computer science field^16^. Some previous studies have attempted deep learning algorithms with knowledge distillation to overcome the modality differences in images^17,18^. Knowledge distillation was introduced by Geoffrey Hinton et al. in Google Inc^19^. Distilling the knowledge is to compress the knowledge into a single model. This comprises first training the cumbersome model called “teacher model” and subsequently using another type of training to transfer knowledge from the cumbersome model to a small model called the “student model”. Since deep learning is outstanding for training multiple levels of feature representation, feature-based knowledge distillation used both the output of the last layer and the output of feature maps in intermediate layers as the knowledge to supervise the student model training^20^.

Based on that craniofacial phenotypes of OSA patients have observable anatomical intrinsic features in the lateral cephalogram, we attempted to develop a fully automated cephalometric screening tool of OSA presence in a simple manner. We aimed to propose a reinforced CNNs algorithm to perform an OSA-classification task in different cephalometric modalities, and to detect the region of interests (ROIs) which indicate the anatomical risk areas contributing to OSA.

## Materials and methods

### Data acquisition with lateral cephalogram protocol

Five hundred lateral cephalograms of adult OSA patients, who had been diagnosed by apnea-hypopnea index (AHI) from PSG record, were randomly collected from picture archiving and communication system (PACS) in Kyung Hee University Medical Center and Dental Hospital. This study samples comprised 100 images (Lateral A) taken with CX-90SP machine (Asahi Roentgen, Kyoto, Japan) and 400 images (Lateral B) taken with DP80P machine (Dentsply Sirona, Bensheim, Germany). The lateral cephalograms were taken in a strictly standardized head and jaw postures at the end of expiration during the respiratory cycle to obtain the most relaxed pharyngeal soft tissue images. The OSA group were subdivided into mild (5 < AHI ≤ 15, n = 100), moderate (15 < AHI ≤ 30, n = 154), and severe (AHI > 30, n = 246) groups according to the disease severity^21^.

For the control group (non-OSA group), 498 cephalograms of healthy orthodontic patients taken with CX-90SP machine (Lateral A) were recruited. Non-OSA samples were defined as the patients without any OSA signs or symptoms in the clinical examination using sleep questionnaire, instead of AHI number. Finally, a total of 998 cephalograms were included in the present study. All patients provided with informed consents for the anonymous use of their cephalograms and PSG. This retrospective study was performed under the institutional review board for the protection of human subject (IRB Number: KH-DT19006).

### Image pre-processing

**Figure 1:**
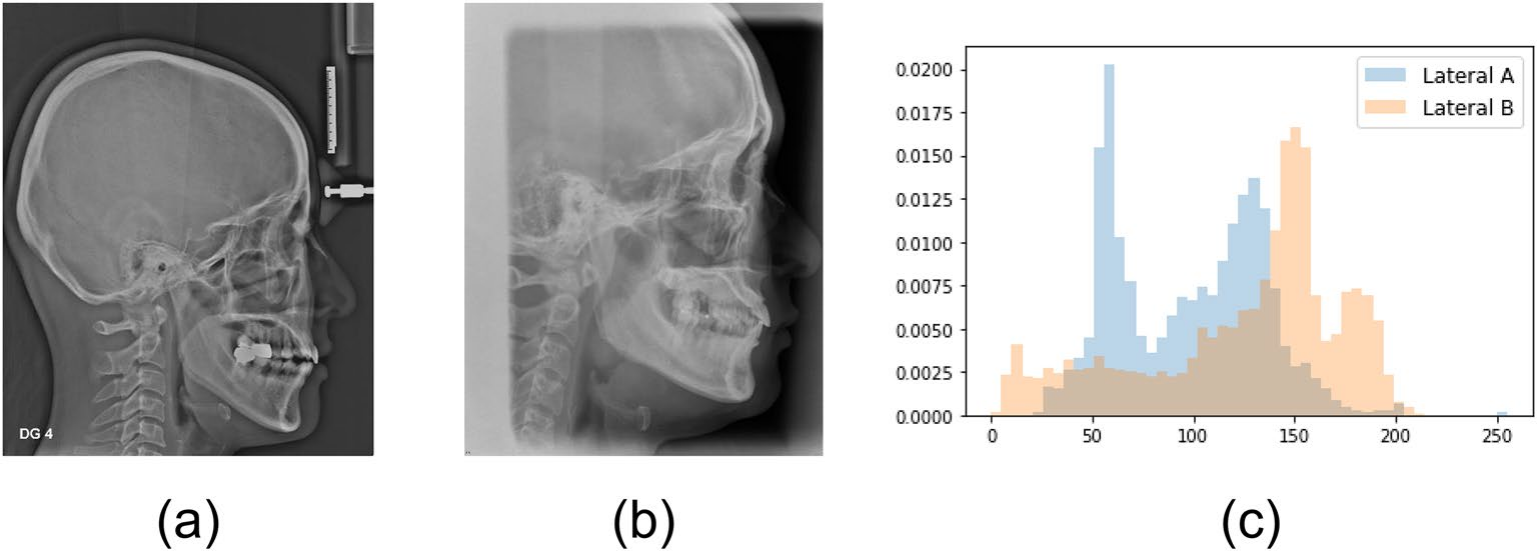
The modality differences between the two lateral cephalograms: (**a**) Lateral A image, (**b**) Lateral B image, and (**c**) histogram distribution of the two images.

**Figure 2:**
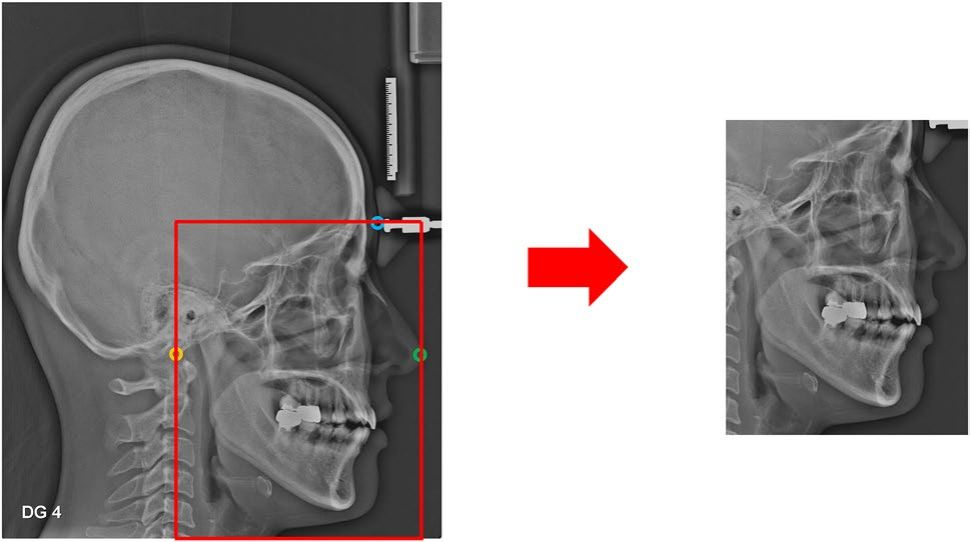
Image cropped based on Landmark. (Yellow: “Basion”, Blue: “Glabella”, Green: “Pronasale”).

The modality differences between Lateral A and Lateral B images existed in the image magnification and resolution as well as the standardization format of image acquisition, which were contrasted by a histogram (Fig. 1). To unify the field of view (FOV) of two image modalities, all images were cropped to include all the potential critical regions of interests (ROIs) predisposing to OSA. Three cephalometric landmarks were automatically extracted with the previously developed landmark-detection algorithm to generate the consistent cropping boundaries: Basion, Glabella, and Pronasale points (Fig. 2)^22^. The accuracy of each landmark selected by algorithm is 0.76 ± 1.06, 1.36 ± 1.08, and 0.60 ± 0.46 mm, respectively^22^. The landmark position selected by algorithm was not modified for cropping. In addition, when resizing to the input shape, the aspect ratio of the original image was maintained to minimize the distortion of the actual image information. For data augmentation, image rotation was applied in a range of − 15° to 15°, and horizontal flip, CLAHE (Contrast Limited Adaptive Histogram Equalization) were applied randomly with a probability of 50%.

### Knowledge distillation

**Figure 3:**
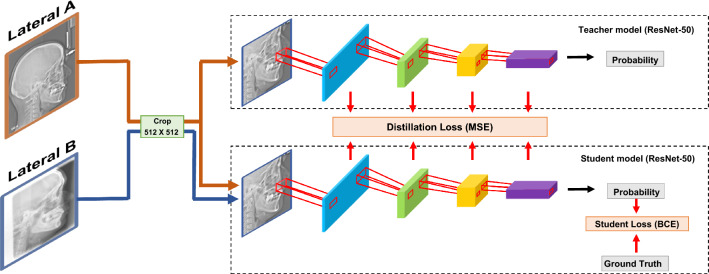
Diagrams of the model architecture.

Since an image of each modality exists predominantly for each group, the regular method is highly likely to create a model that classifies by recognizing the difference in the modality rather than classifying based on rational grounds. Therefore, to overcome this problem, we used a feature-based knowledge distillation deep learning model. Simultaneously, we used ResNet-50 to train the data in order to compare with the proposed model. By applying this method, we (1) first extracted only the modality images common to both groups to create a teacher model, and (2) built a model architecture that ultimately created a student model that additionally learns other modality images while maintaining the performance of this teacher model (Fig. 3). Both models used the ResNet-50 architecture as a backbone, and used pre-trained weights with the ImageNet dataset. Moreover, a comparative experiment was conducted with additional information such as age, and BMI. After global average pooling (GAP) of the ResNet-50 architecture, sex encoded to one-hot vector was added and the combined information passed through the fully connected layers to derive the final OSA prediction values.

### Model training

333 (67%) OSA patient images and 333 (67%) non-OSA patient images were used for training; additionally, 84 (17%) images each from OSA and non-OSA patients were used for validation. For testing, 83 (17%) OSA patient images and 81 (16%) non-OSA patient images were used.

### Evaluation

The performances of the deep neural network models were analyzed for accuracy, area under receiver operating characteristic curve (AUROC), sensitivity, and specificity in classification using Python scikit-learn (version 0.24.2). Unpaired *t* tests were used to compare baseline characteristics between the OSA and non-OSA groups. In addition, paired *t* tests were also performed to compare the results of the model with and without sex. Statistical analysis was performed by Python SciPy (version 1.7.1) in the present study with a two-sided *P* value < 0.05 defined as statistically significant. To verify and visualize the critical ROIs that the proposed deep learning model has seen in prediction the OSA patients from the lateral cephalograms, we performed gradient-weighted class activation mapping (Grad-CAM).

## Results

### Unpaired *t* test between OSA and non-OSA groups

As shown Table 1, the OSA group revealed significantly greater age and higher body mass index (BMI) than non-OSA group. The mean AHI of OSA group was 34.4 ± 22.2 events/h. Unpaired *t* test of the OSA and the non-OSA groups showed significant differences in sex (*p* < 0.01), age (*p* < 0.01), and BMI (*p* < 0.01) between the two groups.

**Table 1:** Baseline demographics of the two groups (OSA, Non-OSA).

Patient characteristics	OSA	non-OSA
n	500	498
Age (years)	45.3 ± 12.5	27.2 ± 8.9
BMI (kg/m^2^)	25.6 ± 3.2	22.2 ± 4.0
AHI (events /h sleep)	34.4 ± 22.2	
Male:female	400:100	205:293

### Analysis through OSA predicted probability values (Fig. 4)

**Figure 4:**
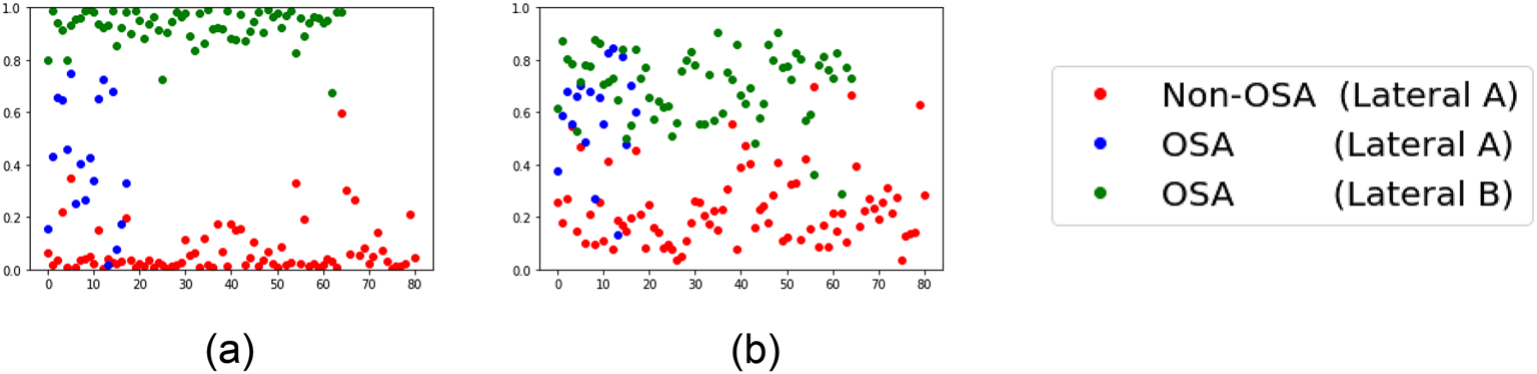
Probability predicted as OSA according to the modality. (a) ResNet-50 Model, (b) Our Model.

In order to check whether the problem of classification by the features appearing in the difference in modality was effectively solved, the OSA prediction probability values of the ResNet-50 and our knowledge distillation models were checked. As shown Fig. 4, the y-axis is the predicted probability value of OSA and the x-axis corresponds to the index number of the data. And the red dot represents the Lateral A image of the non-OSA group, the blue dot is the Lateral A image of the OSA group, and the green dot is the Lateral B image of the OSA group. Through the results of ResNet-50 model (Fig. 4a), it can be confirmed that the red and green dots are predicted with strong certainty as non-OSA and OSA, respectively. On the other hand, the blue dots are evenly distributed over the total probability values. Looking at the result of our model (Fig. 4b), it was observed that red and green dots are generally found to be located on the non-OSA and OSA side, respectively, as they are more evenly distributed than in ResNet-50 model (Fig. 4a), which shows an extreme pattern. Moreover, the blue dot is mostly located on the OSA side.

### Accuracy, AUROC, sensitivity, and specificity of the test set

With respect to the result of the entire dataset, the basic model had the highest values of accuracy, AUROC, and specificity (0.927, 0.982, and 0.988) as shown Table 2. On the other hand, sensitivity had the highest value when cropped in the student model (0.940). In addition, the student model had higher values of accuracy, AUROC, and sensitivity than the teacher model although the specificity was slightly lower.

**Table 2:** The accuracy, AUROC, sensitivity, and specificity of the test set in the binary receiver operating characteristic (ROC) analysis according to the model type.

	Accuracy	AUROC	Sensitivity	Specificity
Base model	0.927	0.982	0.867	0.988
Teacher model	0.841	0.932	0.771	0.914
Student model	0.921	0.965	0.940	0.901

### Analysis of results of our model according to OSA severity

**Figure 5:**
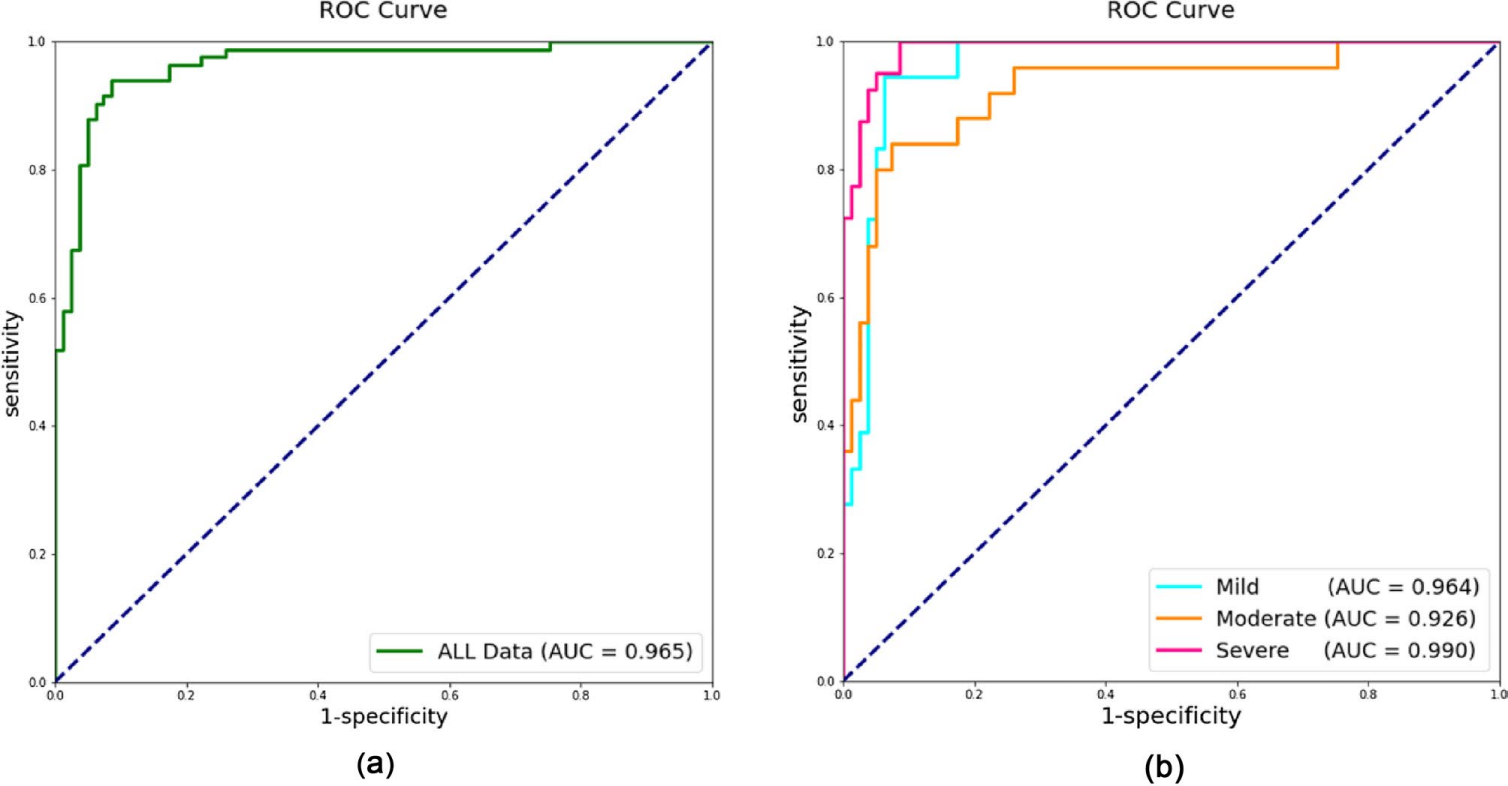
Receiver operating characteristics curves of our model to classify the OSA (**a**) Classifying the OSA and non-OSA. (**b**) Analysis result of OSA according to the severity.

**Figure 6:**
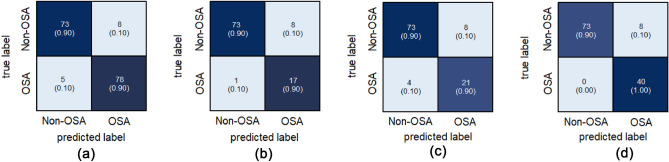
Confusion matrix results of binary classification according to the OSA severity (**a**) all OSA, (**b**) mild, (**c**) moderate and (**d**) severe.

To compare the results according to the OSA severity in our model, we checked the performance of each OSA group in the test set. The severe group (n = 40) showed the highest AUROC (0.990), followed by the mild group (n = 18) and the moderate group (n = 25) (Table ﻿3 and Fig. 5). The results of confusion matrix in each OSA group are shown Fig. 6, respectively.

**Table 3:** Predicted values of our model according to severity of OSA.

Severity	Accuracy	AUROC	Sensitivity	Specificity
Our model				
Mild	0.909	0.964	0.944	0.901
Moderate	0.887	0.926	0.840	0.901
Severe	0.934	0.990	1.000	0.901

### Grad-CAM for diagnosis OSA

**Figure 7:**
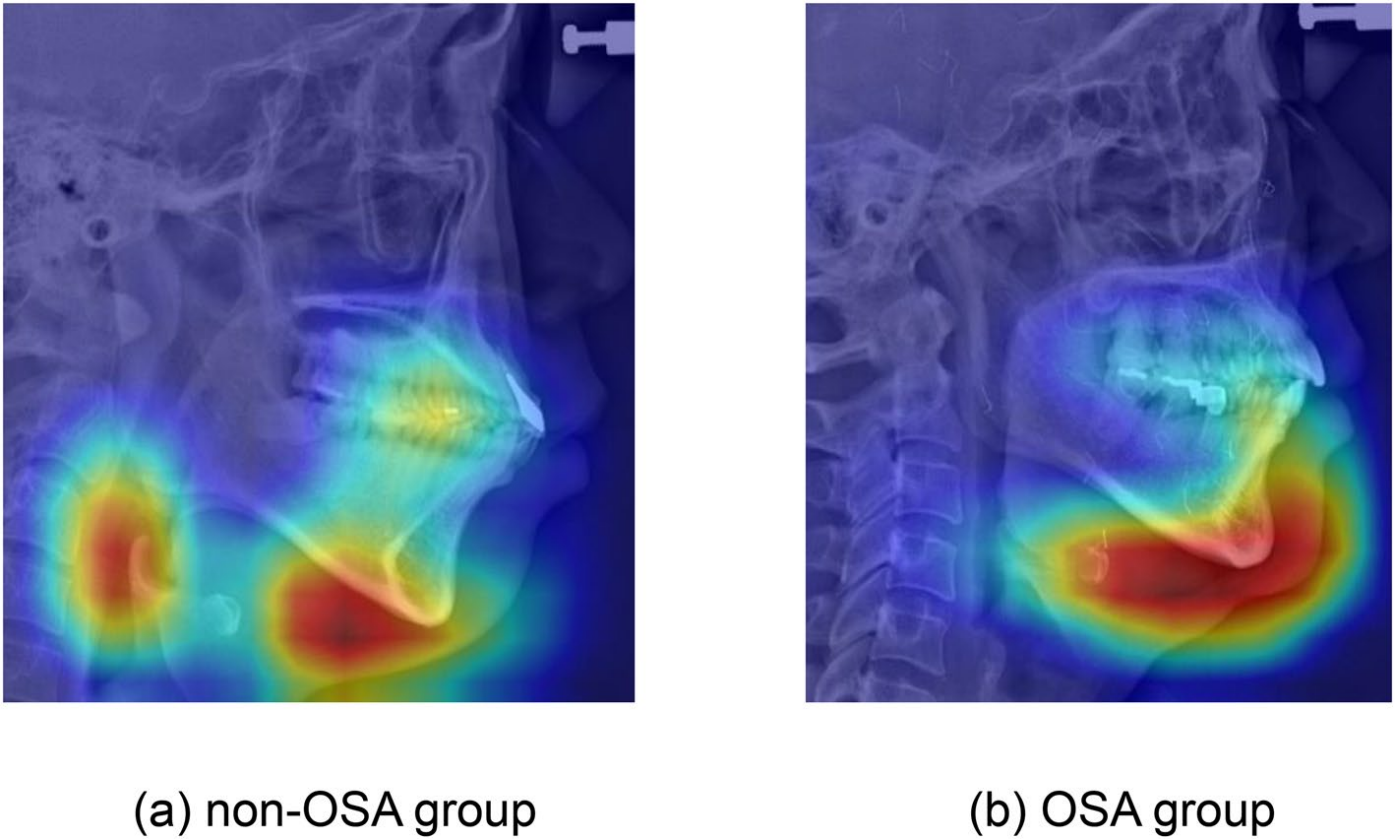
Average result of test set Grad-CAM (**a**) non-OSA group and (**b**) OSA group.

To confirm the results of Grad-CAM in a more objective and universal manner, the results were derived as the average value of all the test sets Table 4. Before applying the average value, registration was applied to all datasets based on landmarks. The results showed different patterns between the OSA and non-OSA groups in Fig. 7. First, in the case of the non-OSA group, our model tends to classify by focusing on the airway and the submental area. On the other hand, our model focused on a wider area, including not only the submental, but also the tip of the chin.

**Table 4:** Comparison of the accuracy, AUROC, sensitivity, specificity of the test set between the models with additional information.

	Accuracy	AUROC	Sensitivity	Specificity
Teacher model	0.841	0.932	0.771	0.914
Student model + sex	0.909	0.962	0.952	0.864
Student model + sex + BMI	0.804	0.792	0.682	0.830
Student model + sex + age	0.822	0.816	0.712	0.860
Student model + sex + BMI + age	0.839	0.828	0.721	0.881

### Comparison of the performance of a classification model with additional information

To confirm the performance change of the model when additional information is included, the performance of the entire test set was compared. The accuracy was higher when learning using only sex than when learning BMI or age together. This might be due to differences in gender differences between the normal group and the OSA group. When Age and BMI were additionally learned, the accuracy was similar to that of the teacher model.

## Discussion

In our study, we proposed a knowledge distillation-based deep learning model to classify the patients into the OSA and non-OSA groups using the lateral cephalogram. Lateral cephalograms have been used as a tool to evaluate the craniofacial morphology in patients with malocclusion in the Department of Orthodontics. Since patients with OSA exhibit anatomical features, which are observable in lateral cephalograms, it is useful to examine whether deep learning can be a screening tool for lateral cephalograms to classify OSA and non-OSA. Tsuiki et al.^14^ attempted to develop a deep learning-based OSA screening tool using the lateral cephalogram for the first time. However, their study did not analyse all OSA severity and included only patients with severe OSA. In the real world, since the imaging device used by each hospital is different, the inevitable differences of image modality should be considered. Therefore, we tried to overcome this problem and developed a robust deep learning model. During the first training using ResNet-50, even though all the images were processed to overcome modality differences, the problem of seeing and classifying OSA with modality still existed because most lateral cephalograms of OSA patients were scanned by a specific imaging modality, DP80P, from the Department of Otolaryngology.

The non-OSA group includes all Lateral A images, whereas the OSA group includes mostly Lateral B images; however, there are also a few Lateral A images. Therefore, by checking the result value of the Lateral A image among the OSA groups, it is possible to evaluate whether the problem caused by the difference in modality has been overcome (corresponding to the blue dot in Fig. 3). Based on the results of the base ResNet-50 model, in the case of blue dots, it was confirmed that they were spread throughout the entire probability values and were particularly biased toward non-OSA. This can be interpreted as the result of the model being classified by recognizing only the same modality information, since the majority of the “Lateral A” corresponds to non-OSA. In contrast, in our model, most of the blue dots are in the OSA group and the green and red dots are also not extremely biased to one side, but rather spread more naturally at a classifiable level; therefore, it can be said that learning was accomplished by overcoming the differences in the modality.

Compared to the model of Tsuiki et al.^14^, our model has slightly higher scores for AUROC, sensitivity, and specificity (Table 3). Although our data are small in number and the difference in modality has significant disadvantages, knowledge distillation overcomes these problems^23^, and the final result is stable and excellent performance is not biased against a specific class compared to the previous study (n = 1389 lateral cephalogram images).

In order to make a more accurate and rational classification model, it is right to learn by adding clinical information other than the images necessary for diagnosing OSA. Therefore, we trained a model with sex information; however, the difference in the performance was insignificant. This is because, first, there is a difference in sex between the two groups, and information about sex could be recognized by itself only through image during training. Therefore, in providing additional information, it is important to select which information to choose, and it is necessary to think about how to provide that information harmoniously with the information of the image.

When discussing the OSA potential, there were four major OSA-related anatomical characteristics that often pay attention which are narrowed pharynx, submental, chin, and nasomaxillary complex. Considering average result of Grad-CAM in Fig. 7, our model focuses on the area around the tip of the chin and the submental area both in the non-OSA and OSA groups. It means that our AI model classified OSA in a way similar to what humans see attentively. In addition, the airway is also an important area in diagnosing OSA. However, unlike the non-OSA group, it is not an area of interest in the OSA group. Normal anatomical features with wide airways can be recognized and classified as normal; however, irregular features of the OSA group, such as narrow or those blurred by the edges of the bones, are considered to be relatively unnoticed in the classification.

There are several limitations in this study. According to Table 1 and the results of the unpaired *t* test, it can be seen that the BMI of the OSA group and the non-OSA group are significantly different, which could lead to classifying by focusing only on the degree of obesity. In fact, it was confirmed that Grad-CAM also focused more on the submandibular fat layer. Moreover, unlike the study by Tsuiki et al.^14^, where only the severe OSA group showing the characteristics of a more crowded oropharynx was tested, we included all the OSA groups in the experiment. Therefore, data with ambiguous characteristics may exist and non-anatomical OSA patients may exist, so it is thought that it was difficult to create an ideal model that can classify by considering all four areas (narrowed pharynx, submental, chin, and nasomaxillary complex) as the OSA areas of interest. For further study, additional clinical information should be considered to train a model with larger sample size. Second, there is no external validation in this study. It is very difficult to acquire an additional external dataset. In the near future, this model should be tested for external validation for evaluation of overfitting and robustness of this model.

## Conclusion

A suitable deep CNN successfully classified the patients with OSA and non-OSA using a 2-dimensional lateral cephalogram. These excellent results were obtained regardless of the severity of the OSA. Even in cephalograms obtained from different X-ray modalities, OSA patients could be accurately identified through appropriate knowledge distillation.

## Data Availability

This retrospective study was conducted according to the principles of the Declaration of Helsinki, and was performed in accordance with current scientific guidelines. The study protocol was approved by the Institutional Review Board Committee of Kyung Hee University Medical Center and Dental Hospital, Korea. (KH-DT19006). The requirement for informed patient consent was waived by the Institutional Review Board Committee of Kyung Hee University Medical Center and Dental Hospital.
